# Multimarker Approach as More Reliable Method Than Single Vitamin D in Relationship with Type 2 Diabetes Mellitus in Montenegrin Postmenopausal Women

**DOI:** 10.3390/biomedicines11102610

**Published:** 2023-09-23

**Authors:** Aleksandra Klisic, Milena Cojic, Dimitrios Patoulias, Ana Ninic

**Affiliations:** 1 Primary Health Care Center, 81000 Podgorica, Montenegro; 2 Faculty of Medicine, University of Montenegro, 81000 Podgorica, Montenegro; 3 Outpatient Department of Cardiometabolic Medicine, Second Department of Cardiology, Aristotle University of Thessaloniki, General Hospital “Hippokration”, 54642 Thessaloniki, Greece; 4 Department for Medical Biochemistry, Faculty of Pharmacy, University of Belgrade, 11000 Belgrade, Serbia

**Keywords:** postmenopausal, vitamin D deficiency, cardiometabolic risk, inflammation, obesity

## Abstract

Objective: Previous studies suggested that ethnic differences, sex and obesity could modify the relationship between 25-hydroxyvitamin D [25(OH)D], glycometabolic markers and/or type 2 diabetes mellitus (T2D). We aimed to examine the potential relationship between [25(OH)D] and T2D in postmenopausal women in Montenegro. In addition, we aimed to explore if a set of biomarkers, rather than [25(OH)D] as a single biomarker, could better explain its potential association with T2D. Patients and Methods: A total of 116 postmenopausal, otherwise healthy women and 48 postmenopausal women with T2D were included. Univariable and multivariable binary logistic regression analysis, along with principal component analysis (PCA), were applied to test the associations between examined biomarkers/set of biomarkers with T2D. Results: Women with T2D had lower serum [25(OH)D] levels than healthy controls (*p* = 0.024). No independent relationship between [25(OH)D] and T2D was found. PCA extracted three significant factors that were associated with T2D, i.e., age-glycometabolic-related factor (i.e., with positive loadings of age, glucose and insulin; OR = 11.321, *p* < 0.001), obesity-inflammation- related factor (i.e., with positive loadings of hsCRP and WC, and negative loading of [25(OH)D]; (OR = 2.079, *p* < 0.001)) and lipid-related factor (i.e., with positive loadings of TG and LDL-c, and negative loading of HDL-c; OR = 1.423, *p* = 0.044). Conclusions: The relationship between [25(OH)D] and T2D is modulated by central obesity (as measured by WC) and inflammation (as measured with hsCRP) in postmenopausal women. Their joint measurement, rather than [25(OH)D] itself, could provide better information for the risk assessment for T2D in postmenopausal women.

## 1. Introduction

Postmenopausal women are at increased risk for cardiometabolic diseases as compared with premenopausal women and with similar risk for the mentioned disorders as men [1]. The increase in androgens in parallel with estrogen decrease, as well as the visceral adipose tissue enlargement, contribute to the increased risk for hypertension, cardiovascular disease, metabolic syndrome, type 2 diabetes mellitus (T2D), non-alcoholic fatty liver disease, etc. [1,2,3,4,5].

Among a variety of biomarkers that were examined in relationship with cardiometabolic risk, vitamin D has taken an important place [6]. Besides its role in bone metabolism, it is attributed to many cardiometabolic properties [1]. We have recently presented the antioxidative and anti-inflammatory potential of 25-hydroxyvitamin D [25(OH)D], the major circulatory form of vitamin D [7], and showed that its supplementation decreased glycated hemoglobin (HbA1c) in T2D [8].

However, results concerning the relationship between [25(OH)D], glycometabolic indices and/or T2D are discrepant [9,10,11,12,13,14]. Serum [25(OH)D] was negatively correlated with homeostasis model assessment of insulin resistance (HOMA-IR) in women, but not in men, and only in those with vitamin D deficiency [9]. On the contrary, another study [10] showed an inverse correlation between [25(OH)D] and HOMA-IR in men but not in women with T2D. These findings implicate the sex-dependent association between serum [25(OH)D] and glycometabolic parameters. Future studies are needed to clarify this issue. Furthermore, ethnic differences are also proposed to modify this relationship [12,13,14]. Serum [25(OH)D] was found to be higher in White than in Black American postmenopausal women [12]. Also, the association between serum [25(OH)D] with HOMA-IR was confirmed in white women but not in Asian or Black postmenopausal women with T2D [13]. Similar findings were confirmed in a US study with a large sample size of non-Hispanic Blacks and Whites [14].

Obesity is another factor that might modulate the relationship between serum [25(OH)D], glycometabolic indices and/or T2D [15,16,17]. It was shown that serum [25(OH)D] was increased after weight loss in T2D-free women with obesity, and the mentioned increase was related to insulin resistance improvement [17].

Considering the complex pathophysiological processes of T2D, a set of biomarkers (i.e., a multimarker approach) rather than a single biomarker could provide more evidence for T2D diagnosis and improve a strategy for prevention of T2D or delay in its complications [18].

Given the fact that previous studies suggested that ethnic differences, sex and obesity could modify the relationship between [25(OH)D], glycometabolic markers and/or T2D, we aimed to examine the potential relationship between [25(OH)D] and T2D in postmenopausal women in Montenegro (i.e., Caucasian women). In addition, we aimed to explore if a set of biomarkers, rather than [25(OH)D] as a single biomarker, could better explain its potential association with T2D.

## 2. Materials and Methods

### 2.1. Study Population

This case-control study encompassed a total of 116 postmenopausal, otherwise healthy women and 48 postmenopausal women with T2D. The participants were included in a consecutive manner when visiting the Primary Health Care Center during their regular check-ups.

The Ethics Committee of the Primary Health Care Center, Podgorica, Montenegro, approved the study protocol, and all postmenopausal women signed informed consent. This study was conducted following the Declaration of Helsinki principles.

Clinical and biochemical data (i.e., after phlebotomy), alongside completed questionnaires (i.e., with history of diseases, history of smoking and alcohol consumption, demographic data and medication use), were collected on the same morning, as explained in detail previously [7]. All postmenopausal women with T2D used metformin.

The inclusion criteria for this study were as follows: women who reported ≥1 year since last menstrual bleeding, with no intake of drugs that might affect the vitamin D level (e.g., vitamin D supplements, anticonvulsants or glucocorticoids), or diseases that could influence circulating vitamin D (e.g., hyperparathyroidism).

Postmenopausal women with a history of autoimmune and malignant diseases, type 1 diabetes mellitus, cardiovascular diseases, liver disease other than steatosis, hypothyroidism, hyperthyroidism, kidney diseases and with high sensitivity C-reactive protein (hsCRP) ≥10 mg/L were excluded from this research. Additionally, women with diabetes mellitus or use of antihyperglycemics were also excluded from the control group.

### 2.2. Biochemical Analyses

The blood samples were collected in the morning after an overnight fast of at least 8 h. Serum levels of fasting glucose, triglycerides (TG), total cholesterol (TC), low-density lipoprotein cholesterol (LDL-c) and high-density lipoprotein cholesterol (HDL-c) were measured spectrophotometrically using standardized enzymatic procedures (Roche Cobas 6000 c 501, Mannheim, Germany). HbA1c and hsCRP were measured with an immunoturbidimetric assay on the same analyzer. Serum levels of [25(OH)D] and insulin were measured by electrochemiluminescence (Cobas 6000/e601, Roche Diagnostics, Mannheim, Germany). HOMA-IR was calculated as follows: HOMA-IR = Fasting glucose (mmol/L) × fasting insulin (µIU/L)/22.5 [7].

### 2.3. Statistical Analysis

Normality of examined data was assessed by Kolmogorov–Smirnov and Shapiro–Wilk’s tests. Differences between the two groups were analyzed by Student *t*-test for normally distributed data and Mann–Whitney U-test for skewed distributed data. Data from these analyses were given as mean ± standard deviation and median (interquartile range), respectively. Categorical data were presented as both absolute and relative frequencies and compared with Chi-square test for contingency tables.

The relationship between glycemic status markers and other tested variables was assessed by non-parametric Spearman’s correlation analysis. The results were given as coefficient of correlation (ρ). The associations of presence of diabetes, given as categorical variable coded as 0—without T2D and 1—with T2D, with [25(OH)D] levels (continuous variable), were analyzed by univariable and multivariable binary logistic regression analysis. Also, univariable binary regression analysis was employed to test the associations of T2D presence and scores obtained from principal component analysis (PCA). The results from these analyses were presented as Odds Ratio (OR) and 95% Confidence Interval (CI). The calculated explained variation in T2D was presented as Nagelkerke R^2^ value.

PCA with varimax rotation was employed to reduce a set of variables into a smaller set of variables or principal components similar to the level of variation. Factor extraction was determined for Eigenvalue larger than 1. The sample adequacy was detected by Kaiser–Meyer–Olkin (KMO) measure for the overall data set, with a measurement larger than 0.5 considered acceptable. The PCA analysis also enables us to calculate scores for factors, which were further used in binary logistic regression analysis.

The statistical analysis was performed using software IBM SPSS version 24.0 (SPSS Corp., Chicago, IL, USA). The results were considered statistically significant for *p* less than 0.05.

## 3. Results

The general data of the tested populations are given in Table 1. Postmenopausal women with T2D were older and had higher body mass index (BMI) and waist circumference (WC) than women without T2D. Also, they were in menopause longer than women without T2D. More postmenopausal women with T2D had hypertension and metabolic syndrome and used cigarettes than women without T2D. Postmenopausal women without T2D had higher concentrations of TC, HDL-c, and LDL-c than postmenopausal women with T2D. Additionally, they had lower levels of TG, glucose, insulin and HbA1c than postmenopausal women with T2D.

**Figure 1:**
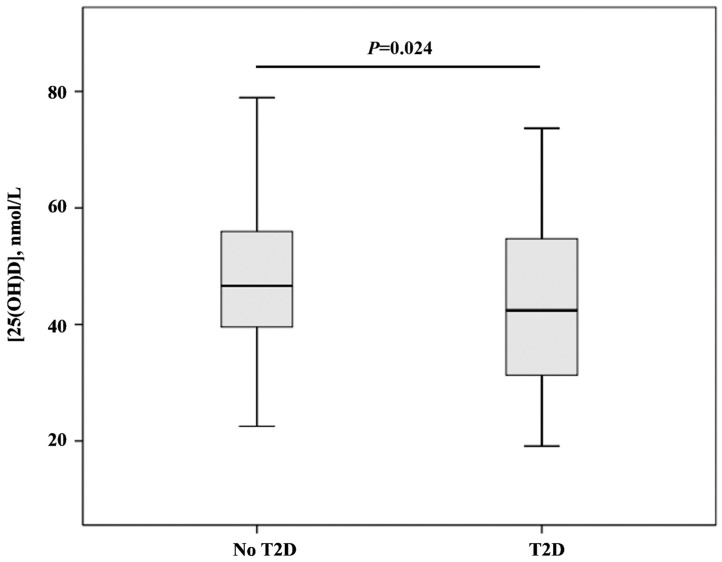
[25(OH)D] levels in groups with and without type 2 diabetes mellitus. Data are given as median (interquartile range) and compared by Mann–Whitney *U*-test.

Serum [25(OH)D] levels were higher in women without T2D [median (interquartile range): 46.63 (39.56–55.96) nmol/L] than women with T2D [median (interquartile range): 42.40 (31.30–54.73) nmol/L] (Figure 1).

Table 2 presents correlation coefficients of glycemic status markers with anthropometric data and biochemical markers in postmenopausal women. Glucose correlated significantly positively with age, BMI, WC, SBP, TG and hsCRP and negatively with TC, HDL-c, LDL-c and [25(OH)D]. Similarly, HbA1c correlated positively with age, BMI, WC, SBP, TG and hsCRP and negatively with HDL-c and [25(OH)D].

Further, we wanted to determine the associations between diabetes and [25(OH)D] in-depth by applying binary logistic regression analysis.

Univariable binary regression analysis demonstrated a significant negative association between diabetes and [25(OH)D], demonstrated by OR = 0.961 (0.934–0.990), *p* = 0.009. Neglkereke R^2^ was 0.064. The multivariable logistic regression analysis with enter selection criteria indicated that [25(OH)D] levels were not independently related to diabetes, when variables that were significantly correlated with glycemic status in Spearman’s correlation analysis (age, WC, HDL-C, TG, hsCRP) were included in the model. The OR was 0.990 (0.952–1.030), *p* = 0.624, and the adjusted R^2^ for the model was 0.556.

PCA was applied to biochemical data to find their relationship with T2D. The sample adequacy was confirmed with the KMO measure (KMO index = 0.703). Bartlett’s test of sphericity was significant (*p* < 0.001). Factors are given in Table 3.

This PCA extracted three significant factors with a total percent of explainable variation of 58% of the investigated parameters. The age-glycometabolic-related factor explained 25% of the variance, and it was associated with positive loadings of age, glucose and insulin levels. The obesity-inflammation-related factor explained 22% of the variance, and it was associated with positive loadings of hsCRP and WC and negative loading of [25(OH)D]. The lipid-related factor explained 19% of the variance, and it was related to positive loadings of TG and LDL-c and negative loading of HDL-c.

Furthermore, we have used scores derived from PCA to estimate the differences between tested groups, i.e., No T2D vs. T2D. The Mann–Whitney test demonstrated that the age-glycometabolic-related factor score was significantly different between the No T2D and T2D groups (*p* < 0.001). Also, a significant difference was determined for obesity-inflammation-related factor score (*p* = 0.002) between the No T2D and T2D groups (Table 4).

Also, we have used scores in univariable binary logistic regression analysis to elucidate possible predictive abilities of factors toward T2D presence (Table 5). The age-glycometabolic-related factor was positively associated with T2D (OR = 11.321, *p* < 0.001). Increased age-glycometabolic-related factor values were associated with more than 11 times greater probability for T2D. This factor explained the variation in [25(OH)D] levels by 60.8%, which was demonstrated by Nagelkerke R^2^ of 0.608. The obesity-inflammation-related factor was positively associated with T2D (OR = 2.079, *p* < 0.001). Increased obesity-inflammation-related factor values were associated with more than two times greater probability for T2D. This factor explained the variation in T2D by 13%, as demonstrated by Nagelkerke R^2^ = 0.130. Finally, the lipid-related factor values were positively associated with T2D (OR = 1.423, *p* = 0.044). Increased lipid-related factor values were associated with a 1.423 times greater probability for T2D. This factor explained the variation in T2D by 3.6%, which was demonstrated by Nagelkerke R^2^ = 0.036.

## 4. Discussion

To our knowledge, this is the first study that investigated the relationship between [25(OH)D] and T2D in postmenopausal women in Montenegro. Although women with T2D exhibited lower serum [25(OH)D] levels than healthy controls, we did not find an independent relationship between [25(OH)D] and T2D in the examined vulnerable population. However, we showed the joint association between [25(OH)D], WC and hsCRP (i.e., obesity-inflammation-related factor) with T2D. The other two sets of biomarkers that were significantly associated with T2D are well-established and include age, glucose, insulin (i.e., age-glycometabolic-related factor), HDL-c, LDL-c and TG (i.e., lipid-related factor).

Multiple biomarkers that explain different metabolic pathways, rather than a single biomarker, may provide deeper insight into the pathophysiological traits of cardiometabolic disorders [18]. In the current study, we found a relationship between [25(OH)D] and glycometabolic indices and T2D. However, only in constellation with higher WC, i.e., abdominal obesity and obesity-related inflammation (hsCRP), the association between [25(OH)D] and T2D became independent.

Muscogiuri et al. [15] showed that fat mass %, sex and BMI were independent predictors of serum [25(OH)D] and confirmed its lower levels in a female than in a male population with no difference in BMI. The authors assumed that a possible explanation for such results could be a higher amount of fat mass in women than in men, given the fact that fat mass represents the storage of fat-soluble [25(OH)D] [19].

In a nationally representative sample of the adult population, Kabadi et al. [16] showed that lower [25(OH)D] and abdominal obesity jointly make an impact on insulin resistance risk. They recorded large magnitude modification of the influence of obesity on the relationship between [25(OH)D] and insulin resistance, i.e., subjects with low [25(OH)D] and obesity exhibited nearly 32 times higher risk for insulin resistance, whereas subjects with sufficient [25(OH)D] and obesity had nearly 20 times higher risk for insulin resistance [16].

Postmenopausal women tend to accumulate fat mass in the visceral compartments, unlike in the premenopausal period [3,5]. In individuals with central obesity, the level of bioavailable vitamin D that activates vitamin D receptors and influences pancreatic b-cells decreases, whereas the risk for poor glycometabolic control increases [19]. Visceral adipose tissue is a major source of adipokines and proinflammatory cytokines that influence signaling pathways of insulin and lead to insulin-resistant state [1,3,5]. Our previous study showed an inverse relationship between hsCRP and [25(OH)D] in healthy postmenopausal women [20]. In the current study, we have confirmed that higher WC and hsCRP jointly with lower [25(OH)D] are independently associated with T2D in postmenopausal women. Accordingly, factorial analysis showed that increased obesity-inflammation-related factor values were associated with more than two times greater probability of T2D onset.

On the other hand, [25(OH)D] by itself exerts an anti-inflammatory role by its ability to attenuate signaling pathways of mitogen-activated protein kinase (MAPK) and to diminish nuclear factor-κB (NF-κB) activation, leading to disruption of pro-inflammatory genes transcription [21]. It was also shown that [25(OH)D] diminishes the transformation of monocytes into macrophages and the secretion of pro-inflammatory cytokines by the latter [22].

Our study lacks the examination of some other biomarkers that were shown to be increased in T2D as compared to prediabetes and healthy controls, such as phospholipids (e.g., sphingomyelin and phosphatidylinositol) and amino acids, especially branched-chain amino acids (e.g., leucine, isoleucine and valine) [23]. A recent meta-analysis [23] that encompassed a total of 34 studies with nearly 3500 T2D patients, 14,500 healthy individuals and 2140 patients with pre-diabetes showed that some amino acids (i.e., proline, valine, lysine, isoleucine, leucine, glutamate and tyrosine) were higher whereas glycine was lower in T2D as compared to healthy controls. They also recorded the difference in levels of palmitic acid, glutamate and alanine between patients with pre-diabetes and T2D. In light of these facts, future studies are needed to extend the spectrum of biomarkers that would be beneficial for early diagnosis of (pre)diabetes and that could be reliable in monitoring the disease progression.

This study is also limited by its cross-sectional nature and, thus, cannot confirm the causality between examined factors and T2D. A small sample size might affect the results. The inability to identify and quantify the metabolites with liquid chromatography–mass spectrometry (LC–MS), a more reliable technique, is another limitation of this study. Additionally, we were limited in precise imaging methods to measure visceral compartments, and we used waist circumference as a proxy of central obesity. Moreover, we have included only a cohort of postmenopausal women. Hence, future studies are needed to include both genders and women of reproductive age. Since postmenopausal status was not confirmed by sex hormones measurement but by self-report of participants, recall bias cannot be excluded. Nevertheless, this is the first study that investigated the relationship between [25(OH)D] and T2D in postmenopausal women in Montenegro and one of the rare studies that applied multimarker assessment to gain deeper knowledge in the clarification of different pathophysiological mechanisms of T2D in postmenopause. Longitudinal and nationally representative studies are needed to support the obtained results.

## 5. Conclusions

We have presented three sets of biomarkers that were significantly associated with T2D in postmenopausal women, i.e., age, glucose and insulin (i.e., age-glycometabolic-related factor), HDL-c, LDL-c and TG (i.e., lipid-related factor) and WC, hsCRP and [25(OH)D] (i.e., obesity-inflammation-related factor). Given the obtained results of this study, we assume that the relationship between [25(OH)D] and T2D is modulated by central obesity (as measured by WC) and obesity-related inflammation (as measured by hsCRP) in postmenopausal women. Their joint measurement, rather than [25(OH)D] by itself, could provide a better evaluation of the risk for T2D. The measurement of this set of parameters might be a good strategy for prevention and/or timely diagnosis of T2D in postmenopausal women.

## Figures and Tables

**Table 1:** General characteristics of populations with and without type 2 diabetes mellitus.

Variable	No T2D	T2D	*p*
Postmenopausal women, N	116	48	
Age, years	57.22 ± 4.66	63.15 ± 7.46	<0.001
BMI, kg/m^2^ *	25.55 (22.85–28.25)	29.43 (26.35–32.79)	<0.001
WC, cm *	86.5 (78.0–95.0)	99.5 (94.0–105.5)	<0.001
SBP, mmHg *	129 (110–147)	133 (121–143)	0.142
DBP, mmHg *	84 (70–95)	79 (73–83)	0.191
Hypertension, N (%)	45 (38.8)	48 (100)	<0.001
Smoking status, N (%)	0 (0)	16 (33.3)	<0.001
Menopause duration, years	5 (2–10)	14 (8–19)	<0.001
MetS, N (%)	31 (26.7)	48 (100)	<0.001
Diabetes duration, years	-	6 (3–8)	-
TC, mmol/L	6.48 ± 1.06	5.66 ± 1.27	<0.001
HDL-c, mmol/L	1.72 (1.40–2.00)	1.37 (1.15–1.55)	<0.001
LDL-c, mmol/L	4.11 (3.60–4.80)	3.35 (2.78–4.19)	<0.001
TG, mmol/L *	1.19 (0.90–1.68)	1.78 (1.31–2.26)	<0.001
Glucose, mmol/L *	5.2 (5.0–5.55)	7.5 (6.60–8.35)	<0.001
Insulin, μIU/L *	5.89 (4.28–7.98)	11.72 (8.56–16.53)	<0.001
HsCRP, mg/L *	0.93 (0.43–1.97)	1.65 (0.69–3.03)	0.001

Normally distributed variables are given as mean ± standard deviation and compared by Student *t*-test. * Skewed distributed variables are presented as median and interquartile range and compared by Mann–Whitney *U*-test. Categorical variables are presented as absolute and relative frequencies and compared with Chi-square test for contingency tables.

**Table 2:** Spearman’s correlation coefficients of glycemic status markers and clinical data in individuals with and without type 2 diabetes mellitus.

Variable	Glucose, mmol/L		HbA1c, %	
	ρ	*p*	ρ	*p*
Age, years	0.415	<0.001	0.404	<0.001
BMI, kg/m^2^	0.427	<0.001	0.642	<0.001
Waist circumference, cm	0.497	<0.001	0.682	<0.001
SBP, mmHg	0.299	<0.001	0.390	<0.001
DBP, mmHg	0.116	0.147	0.229	0.004
Menopause duration, years	0.076	0.416	0.098	0.298
TC, mmol/L	−0.201	0.010	−0.130	0.097
HDL-c, mmol/L	−0.379	<0.001	−0.337	<0.001
LDL-c, mmol/L	−0.168	0.031	−0.111	0.156
TG, mmol/L	0.383	<0.001	0.332	<0.001
HsCRP, mg/L	0.257	0.001	0.401	<0.001
[25(OH)D], nmol/L	−0.175	0.025	−0.302	<0.001

Data age given as coefficients of correlation Rho (ρ).

**Table 3:** Factors related to type 2 diabetes mellitus and extracted by principal component analysis with percent of variability and variables’ loadings.

Factors	Variables (Loadings)	Factor Variability
Age-glycometabolic-related factor	Age (0.846) Glucose (0.767) Insulin (0.658)	25%
Obesity-inflammation-related factor	HsCRP (0.801) WC (0.746) [25(OH)D] (−0.623)	22%
Lipid-related factor	TG (0.826) HDL-c (−0.698) LDL-c (0.634)	19%

**Table 4:** Differences in factor scores between groups with and without type 2 diabetes mellitus.

Factors	*p* for the Difference between No T2D and T2D
Age-glycometabolic-related factor	<0.001
Obesity-inflammation-related factor	0.002
Lipid-related factor	0.050

**Table 5:** Odds ratios (OR) after univariable binary logistic regression analysis for factors predicting abilities towards type 2 diabetes mellitus presence.

Predictors	Unadjusted OR (95%CI)	*p*	Nagelkerke R^2^
Age-glycometabolic-related factor	11.321 (5.369–23.874)	<0.001	0.608
Obesity-inflammation-related factor	2.079 (1.377–3.137)	<0.001	0.130
Lipid-related factor	1.423 (1.010–2.005)	0.044	0.036

## Data Availability

The data will be available upon reasonable request (contact person: aleksandranklisic@gmail.com).
